# Niclosamide combined to Azacitidine to target *TP53*-mutated MDS/AML cells

**DOI:** 10.1038/s41375-024-02281-z

**Published:** 2024-05-22

**Authors:** Nabih Maslah, Salome Rety, Melina Bonnamy, Lorea Aguinaga, Tony Huynh, Veronique Parietti, Stephane Giraudier, Pierre Fenaux, Bruno Cassinat

**Affiliations:** 1 INSERM UMR 1131, Universite Paris Cite, IRSL, Paris, France; 2 APHP, Hopital Saint-Louis, Laboratoire de Biologie Cellulaire, Universite Paris Cite, Paris, France; 3 APHP, Hopital Saint-Louis, Service d’Hematologie Seniors, Universite Paris Cite, Paris, France; 4 Université Paris Cité, INSERM/CNRS, US53/UAR2030, Institut de Recherche Saint-Louis, Paris, France

**Keywords:** Preclinical research, Translational research, Haematological cancer

## To the Editor:

Myelodysplastic syndromes (MDS) are malignant bone marrow disorders characterized by ineffective hematopoiesis leading to refractory cytopenias and an increased risk of progression to acute myeloid leukemia (AML). Prognosis of those diseases is stratified on the basis of the percentage of blasts in the bone marrow, karyotype and the number of cytopenias according to a revised International Prognostic Scoring System (IPSS-R), recently revised by the incorporation of somatic mutations [1]. *TP53* gene mutations occur in 5–10% of all MDS and AML cases [2], including 20–25% of the low-risk MDS with isolated del 5q, where they are often monoallelic [3] and 40–50% of MDS and AML with complex karyotypes, where they are generally biallelic [4]. In the latter group, presence of *TP53* mutation is generally associated with resistance to all treatments available, including hypomethylating agents (HMAs, including azacytidine-AZA) and allogeneic stem cell transplantation, and a very poorer outcome [5]. Consequently, drugs targeting mutant p53 are in active development including the p53 reconforming agent APR-246 in association to AZA, which showed promising results in vitro [6, 7] and in a phase 2 study, however not confirmed in a phase 3 study comparing AZA+APR-246 and AZA alone.

Niclosamide (NCL) is an oral salicylanilide derivative approved worldwide since 1960 for the treatment of human intestinal tapeworm infections [8, 9]. It is a hydrogen ionophore that translocates protons across the mitochondrial membrane resulting in mitochondrial uncoupling and futile cycles of glucose and fatty acid oxidation. Reports suggest that NCL inhibits tumor growth promoting pathways, including WNT/beta-catenin, STAT3, Notch, and mTOR pathways, although its exact antitumor mechanism is not entirely clear. In hematological malignancies NCL has shown effectiveness in T-acute lymphoblastic leukemia [10] and chronic myeloid leukemia [11] through various mechanisms of action, while it induces cell death in AML through the modulation of CREB pathway [12] or LEF transcription factor [13]. Recently, in solid tumor models, the NCL-induced mitochondrial uncoupling was shown to preferentially impair the proliferation of p53-knock-out cells and of p53 mutant patient-derived ovarian xenografts [14].

**Fig. 1:**
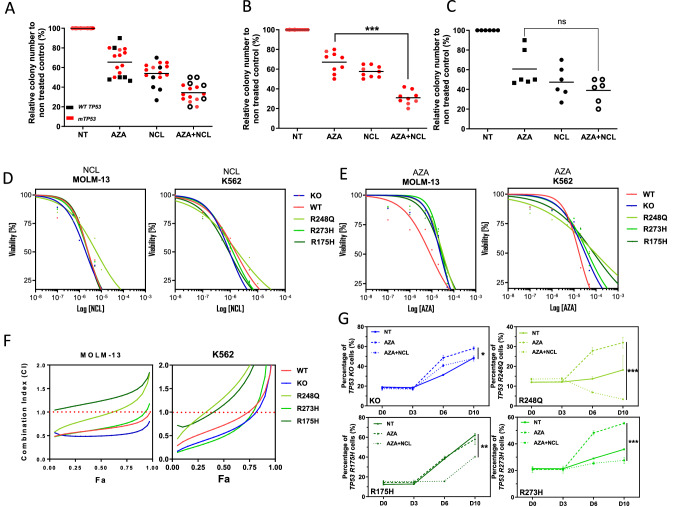
Niclosamide associated with Azacitidine targets *TP53*-mutated cells MDS/AML in vitro. **A** Relative colony numbers to non-treated (NT) control of *n* = 15 primary MDS/AML treated with Niclosamide (NCL), Azacitidine (AZA) or the combination of both drugs (AZA + NCL) at day 14 of culture. Red dots: *TP53*-mutated (m*TP53*) samples. Black dots: *TP53* wild-type (WT *TP53*) samples. **B** *TP53*-mutated samples only. **C** *TP53* WT samples only. **D** Dose effect curves of isogenic *TP53* WT or mutated (KO, R248Q, R273H and R175H) MOLM-13 and K562 to NCL. **E** Dose effect curves of isogenic *TP53* WT or mutated (KO, R248Q, R273H and R175H) MOLM-13 and K562 to AZA. **F** Combination Index (CI) calculated for the association of AZA + NCL at *n* = 10 different concentrations according to Chou TC et al. [18]. Theoretical Basis, Experimental Design, and Computerized Simulation of Synergism and Antagonism in Drug Combination Studies, Pharmacological Rev. 2006 Sep;58(3):621-81. doi: 10.1124/pr.58.3.10. **G** Evolution of GFP-positive *TP53*-mutated or KO cells from day 0 (D0) to day 10 (D10) at each condition of treatment (NT, AZA alone at IC30 for the mutated cell line or AZA + NCL at IC30). All experiments have been performed at least twice in triplicate. *p < 0.05, **p < 0.01, ***p < 0.001.

In this study, we aimed at testing whether NCL alone or in combination with AZA could preferentially affect *TP53*-mutated over *TP53* -WT MDS/AML cells. First, we measured the clonogenic potential of primary MDS/AML cells using previously described culture conditions [6]. AZA and NCL alone inhibited the clonogenic growth (AZA: Inhibition of colony growth: 35.4%, NCL: 44.8%, Fig. 1A*p* < 0.01 vs. NT). Remarkably, the combination of the two drugs strongly decreased the clonogenic growth of leukemic cells (AZA + NCL: 66.2% vs. AZA alone: 35.4%, *p* < 0.0001) suggesting an additive or synergistic anti-leukemic potential of the combination. When patient samples were analyzed according to the *TP53* genotype, the combination appeared to be more effective on *TP53*-mutated (m*TP53*) samples (Difference between AZA + NCL vs. AZA in WT: 21.8% *p* = ns and in m*TP53*: 35.1% *p* < 0.001, Fig. 1B, C). Of the 9 *TP53* mutated patients tested 8 had missense point mutations while only 1 patient harbored a truncating mutation (Table S1) making it difficult to conclude on the differential effect of the drug according to the type of TP53 mutation.

To further explore the selectivity of NCL in AML cells according to *TP53* status, we determined the sensitivity to this drug in two isogenic AML models MOLM-13 and K562. We tested both wild type *TP53* parent cells and isogenic cells engineered to a *TP53* knock-out (KO) or various *TP53* mutations (p.R175H, p.R248Q or p.R273H) [15]. The NCL concentration inhibiting 50% of the proliferation (IC50) was found slightly lower in *TP53* KO cells compared to wild type cells (IC50 MOLM-13 KO: 1.9 uM vs. MOLM-13 WT: 2.6 uM, *p* < 0.05, Figs. 1D and S1A, B) as previously described in other models [14]. Compared to WT cells, mutated cell lines did not show a higher sensitivity to NCL, some even having a slightly higher IC50 (IC50 MOLM-13 R248Q: 5.9 uM vs. IC50 MOLM-13 WT: 2.6 uM, *p* < 0.01, Figs. 1D and S1A, B). These results suggested a higher sensitivity of *TP53* KO cells to NCL compared to WT cells and *TP53*-mutated cells (which are hemizygous for the mutation). The sensitivity of *TP53*-mutated cell lines to AZA after 72 h of culture was found on average 4 times lower compared to wild-type cell lines, confirming recent results [16] (IC50 AZA MOLM-13 WT: 8.6 uM vs. MOLM-13 R248Q: 32.3 uM, *p* < 0.001, Figs. 1E and S1A, B). However, addition of low doses of NCL (IC20) to AZA (IC50) significantly reduced the proliferation of the *TP53*-KO MOLM-13 and K562 cells compared to AZA alone, while the *TP53* WT cells were affected to a lesser extent (Fig. S1C). We then studied the proliferation of different *TP53*-mutated MOLM-13 cells and observed that both R248Q- and R273H-mutated cells were more sensitive to the combination (IC50 AZA R248Q: 33.6 uM vs. IC50 AZA + IC20 NCL R248Q: 9 uM, *p* < 0.0001 Fig. S1D) compared to WT cells (IC50 AZA WT: 7.1 uM vs. IC50 AZA + IC20 NCL WT: 5.8 uM, *p* = ns Fig. S1D). Of note, R175H-mutated cell lines appeared to only weakly respond to the combination (IC50 AZA MOLM-13 R175H: 28.3 uM vs. IC50 AZA + IC20 NCL MOLM-13 R175H: 21.4 uM, *p* = ns, Fig. S1D) suggesting a selectivity of action on specific mutation subtypes. Similar results were obtained using K562 cells harboring the same *TP53* genotypes as MOLM-13 cells (Fig. S1C, D). These data were further confirmed in a combination index (CI) analysis which allows to test the synergy between two drugs for a Fraction affected (Fa) equal to 0.5 as described previously (see Supplementary experimental procedures). By testing ten concentrations (of each drug in combination) on *TP53* wild type and mutated MOLM-13 cell lines, we found a synergistic effect for every cell line, including *TP53* WT cells (Combination Index at Fa = 0.5 MOLM-13 R273H: 0.61, Fig. 1F) with the exception of the R175H-mutated line. In the presence of this particular mutation, the combination was antagonistic whatever the doses used (Combination Index at Fa = 0.5 MOLM-13 R175H: 1.23, Fig. 1F). This synergistic effect was confirmed in the K562 isogenic cell lines at low concentrations but not at high concentrations, probably reflecting cell line heterogeneity driven by different oncogenes.

To further confirm the selective effect of NCL and its combination with AZA on *TP53*-mutated cells, we mixed MOLM-13 WT cells (labeled with mCherry) with each MOLM-13 *TP53*-mutated cells (labeled with GFP). AZA treatment significantly increased the proportion of *TP53*-mutated cells for R248Q and R273H mutations while no difference was observed with R175H-mutated cells (Fig. 1G). Reasons for the differential response in those various mutations genotype are not clear. It has been suggested that some drugs like Arsenic Trioxide could reconform the mutant protein partially or totally depending on the missense mutant subtype [17]. NCL is believed to target *TP53* mutated cells through a mitochondrial uncoupling [14] which may be differently altered by those various mutations. When low concentrations of NCL were added to AZA, we observed a strong decrease in the proportion of *TP53*-mutated cells and an increase of *TP53* WT cells whatever the mutation tested (Fig. 1G), confirming preferential targeting of *TP53*-mutated cells by the combination of AZA + NCL in vitro. However, the effect of the AZA + NCL combination in *TP53* KO cells was weaker than in mutant cells. Kumar et al. [14] showed that NCL impaired the oxphos chain preferentially in *TP53* KO cells compared to WT cells. To explain the better sensitivity of mutated cells one could suggest that those cells may rely more than KO cells on mitochondria to survive. NCL treatment would therefore induce greater mitochondrial apoptosis in missense mutant than in KO cells.

**Fig. 2:**
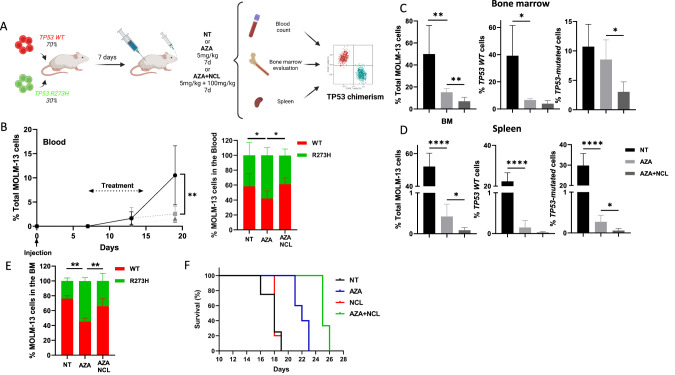
In vivo combination of Azacitidine and Niclosamide allows clearance of *TP53*-mutated cells in a MDS/AML xenograft model. **A** Diagram of the in vivo experimental procedure. MOLM-13 *TP53* WT-mCherry and *TP53* R273H-GFP were mixed at a ratio of 70–30% and injected to NSG mice (D0). At day 7 mice were randomly assigned to the non-treated (NT, *n* = 4), Azacitidine alone (AZA, *n* = 5) or Azacitidine + Niclosamide (AZA + NCL, *n* = 5) group and were treated at the dose and during the time described. Evaluation of treatment efficacy was performed on the total cells (%mCherry + %GFP) and on *TP53* chimerism every week in the blood and at sacrifice in the Bone marrow, Spleen and Blood. **B** Percentages of total MOLM-13 cells, *TP53* WT only or *TP53*-mutated only MOLM-13 cells in blood over time under indicated treatments. Percentages of total MOLM-13 cells, *TP53* WT only or *TP53*-mutated only MOLM-13 cells in (**C**) bone marrow and (**D**) Spleen at day 19 (end of the experiment) at each condition of treatment. **E** *TP53* chimerism in the bone marrow (BM) at the end of the experiment at each condition of treatment NT, AZA or AZA + NCL. **F** Survival analysis on a separate group of NSG mice injected as described in (**A**) and treated with AZA 2.5 mg/kg/j IP for 7 days and NCL 100 mg/kg/j for 14 days. *p < 0.05, **p < 0.01, ***p < 0.001, ****p < 0.0001.

To further explore the efficacy of the AZA + NCL combination in vivo, we injected a mixture of 30% R273H-mutated cells (GFP) and 70% WT (mCherry) in NSG mice (Fig. 2A). In the absence of treatment, mice developed clinical signs approximately 3 weeks after injection with massive blast infiltration of bone marrow (BM), blood and spleen (Fig. S2A–C). As expected, AZA treatment efficiently inhibited tumor infiltration of total MOLM-13 cells in the BM, spleen and blood (BM MOLM-13 infiltration in NT: 48.3% vs. AZA: 17.5%, *p* < 0.01; Spleen infiltration in NT: 52.1% vs. AZA: 0.4%, *p* < 0.0001, Fig. 2B–D). However, the proportion of *TP53*-mutated cells in the BM increased significantly with AZA treatment (% R273H-GFP cells in NT: 31.3% vs. AZA: 54.8%, *p* < 0.05, Fig. 2E). The combination of NCL to AZA induced a massive clearance of leukemic cells in all compartments (BM infiltration in AZA: 17.5% vs. AZA + NCL: 8.1%, *p* < 0.01; Spleen in AZA: 0.42% vs. AZA + NCL: 0.08%, *p* < 0.0001, Fig. 2B–D). Strikingly, with AZA + NCL treatment, we observed a decrease in the proportion of *TP53*-mutated cells in the BM as compared to AZA treatment alone (%R273H-GFP in AZA: 54.8% vs. AZA + NCL: 34.1%, *p* < 0.05, Fig. 2E). Finally, using the AZA + NCL combination in vivo we observed a trend to a better survival in NSG mice injected with the mix of MOLM-13 cells described above (Fig. 2F).

MDS/AML patients with *TP53* mutations represent a poor prognosis group in which AZA therapy is largely ineffective, mostly due to a resistance of *TP53*-mutated cells [16]. Using Niclosamide in combination with AZA on two different AML models, we found a higher efficacy compared to AZA alone. More interestingly, we observed that NCL suppressed the AZA-induced selection of various *TP53*-mutated cells suggesting it could restore the sensitivity of *TP53*-mutated cells to hypomethylating agents. We are now considering using this combination in the treatment of MDS/AML patients with TP53 mutation.

### Supplementary information

Supplementary data
