# Supplementary material for: Niclosamide combined to Azacitidine to target TP53-mutated MDS/AML cells
Source: Leukemia. 2024 May 22;38(7):1630–3. doi: 10.1038/s41375-024-02281-z (PMC11216995; doi:10.1038/s41375-024-02281-z)
Supplement: Supplementary file 1 — Supplementary data [file 41375_2024_2281_MOESM1_ESM.pdf]

Supplementary Methods

Reagents and drugs

Niclosamide (NCL) and AZA were purchased from Sigma Aldrich (Summit, NJ, USA) and were kept frozen at -80°C as DMSO stock solutions.

Cell lines and primary cells

The AML cell lines MOLM-13 and K562 (kindly provided by Steffen Boettcher, University Hospital of Zurich, Switzerland) were previously characterized (Boettcher *et al*, 2019) and CRISPR modified to introduce several different *TP53* genotypes. Before starting experiments, the *TP53* status was confirmed for each individual clone using a 36 genes NGS panel (Sophia Genetics) exploring the full *TP53* gene sequence. We confirmed the presence of a knock-out (KO) or hemizygous mutation of *TP53* for each genotype (p.R175H, p.R248Q and p.R273H). Cell lines were grown in RPMI 1640 medium (GIBCO; Life Technologies Corporation, Carlsbad, CA, USA) supplemented with 20% FBS (GIBCO) (MOLM-13 cells) or 10% FBS (K562 cells), 1% Penicillin/Streptomycin (P/S), and 1% GlutaMAX. Cells in the logarithmic phase of growth that had been seeded at a density of $3x10^5$/mL were used for all of the experiments.

Primary cells from MDS and AML patients known to have mutated *TP53* were provided by the Service d'Hematologie of the Hôpital Saint-Louis, Paris, France, after informed consent had been received. Also, primary cells were isolated from healthy blood donors as controls. In both cases mononuclear cells were isolated using a Ficoll gradient centrifugation. This study was approved by the Groupe Francophone des Myélodysplasies (GFM) review board.

Proliferation assay

The following concentrations were used for each drug: NCL from 10^{-8} M to 10^{-5} M and AZA from 10^{-8} M to 5.10^{-5} M. Proliferating cells were plated, incubated for three days, and then counted. As previously described (Maslah *et al*, 2020) both drugs were added once at day 0 of cell culture and

cytotoxicity was evaluated after 72h of culture. Viability was assessed by Trypan blue exclusion. Experiments were performed at least twice in triplicate. Synergistic studies were performed using Compusyn (Combosyn) Software (Chou, 2006) which allowed the Combination Index (CI) calculation according to the following expression:

CI = [Drug1 in combo]/ [Drug1 alone] + [Drug2 in combo]/[Drug2 alone]

Clonogenic assays

Due to partial blast infiltration in the majority of samples, as generally reported in MDS/AML with a complex karyotype and *TP53* mutation, we used semi-solid cultures to test for the impact of drugs on progenitor clonogenicity. Briefly, $3x10^5$ peripheral blood mononuclear cells (PBMC) or $1x10^5$ bone marrow-derived mononuclear cells (BMMC) were cultured in cytokine-containing methylcellulose (MethoCult™, 84434, STEMCELL Technologies) in the presence of the IC50 of each drug (1 and 3 uM NCL or 3 uM AZA) alone or in combination as previously described (Maslah *et al*, 2020). After 14 days at 37°C, the erythroid and myeloid colonies were counted.

Statistical analysis

All experiments were performed at least twice in triplicate. The results are expressed as means±standard deviation (SD). All of the single-parameter measurement comparisons were determined using the Mann-Whitney test (PRISM 18964 software, GraphPad, La Jolla, CA, USA). All tests were two-sided; $p<0.05$ was considered statistically significant. Combination indices were calculated by CompuSyn software according to the Chou-Talalay method for drug synergy studies (Chou, 2006).

Competition assays

5.10^4 cells composed of a mixture of 70% of MOLM-13 *TP53* WT-mCherry and 30% of *TP53* KO-GFP, R248Q-GFP, R273H-GFP or R175H-GFP were cultured for 10 days and treated or not with AZA, NCL or

AZA+NCL. *In vitro* chimerism was performed at day 3, 6 and 10 under each condition of treatment by flow cytometry (Cytoflex, Beckmann Coulter).

Murine models

NSG mice were provided by the Institut de Recherche Saint-Louis (IRSL) animal facility, Hôpital Saint-Louis. A total of 5.10^5 cells made of 70% of MOLM-13 *TP53* WT-mCherry and 30% of MOLM-13 *TP53* R273H-GFP were injected to 12 weeks old NSG mice and assigned randomly to non treated (NT), AZA, NCL or AZA+NCL group. AZA treatment was administered intraperitoneally at 5 mg/kg and NCL by oral gavage at 100 mg/kg. Mice were all sacrificed at day 19 when clinical signs of disease appeared.

References

Chou, T.-C. (2006) Theoretical basis, experimental design, and computerized simulation of synergism

Characteristics of TP53-mutated patients

UPN	TP53 mutation	p53 protein expected expression	VAF (%)	Clonogenic effect AZA (%)	Clonogenic effect NCL (%)	Clonogenic effect AZA+NCL (%)
#1	p.278R	persistent expression	73%	25.00	35.00	71.88
#2	p.R196X	no expression	33%	50.00	47.50	75.00
#3	p.H179L	persistent expression	47%	28.20	48.00	72.40
#4	p.M237I	persistent expression	81%	38.00	37.80	66.85
#5	p.V132R	persistent expression	16%	45.50	42.50	58.00
#6	p.V173L<br>p.P278T	persistent expression	24%<br>22%	22.00	40.00	80.00
#7	p.P151S<br>p.P278S	persistent expression	36%<br>28%	27.50	45.50	67.50
#8	p.V216M	persistent expression	50%	20.00	35.00	60.00
#9	p.P151R	persistent expression	38%	21.00	39.00	61.50

Supplementary Figure 1

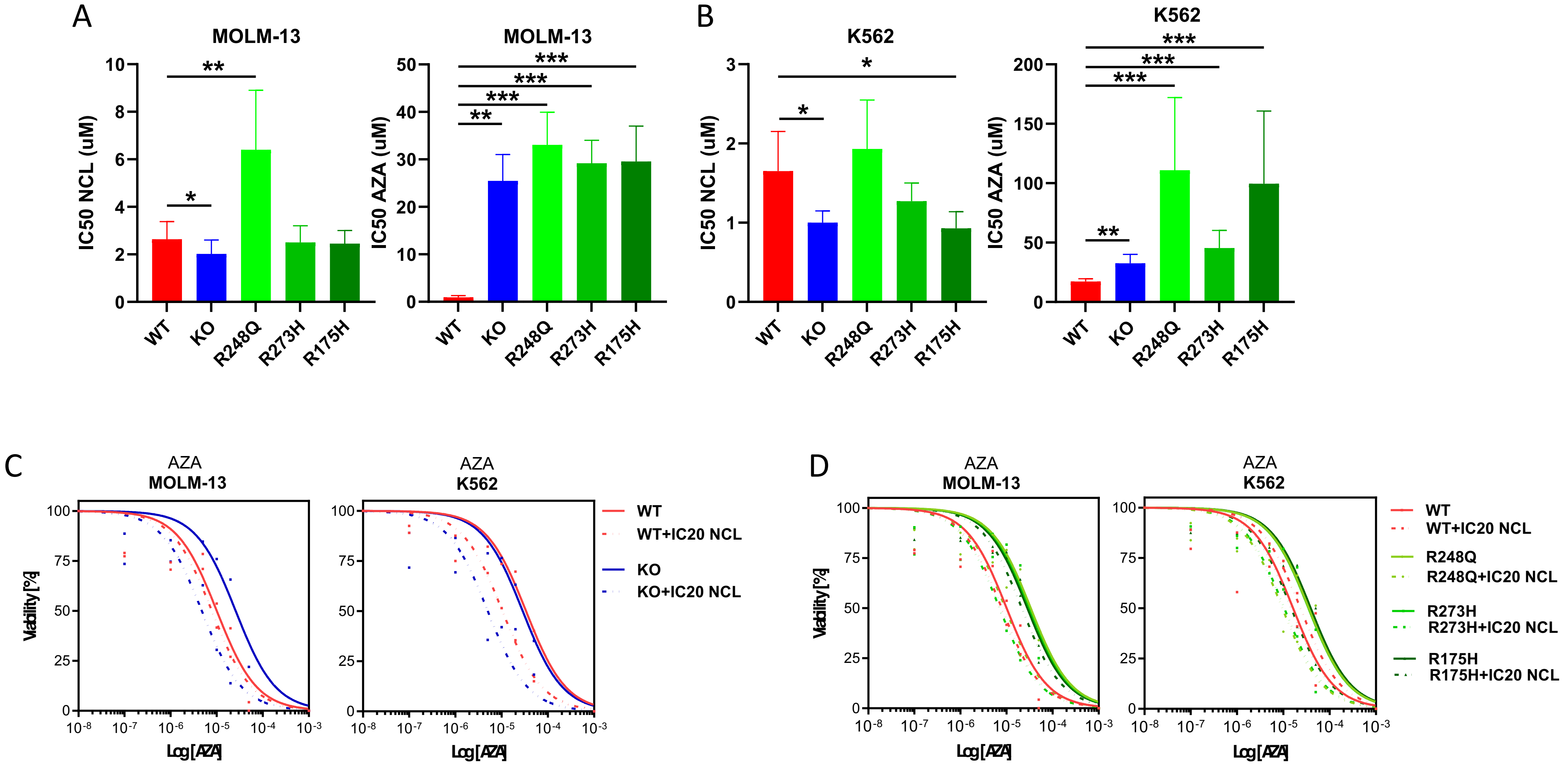

Supplementary Figure 2

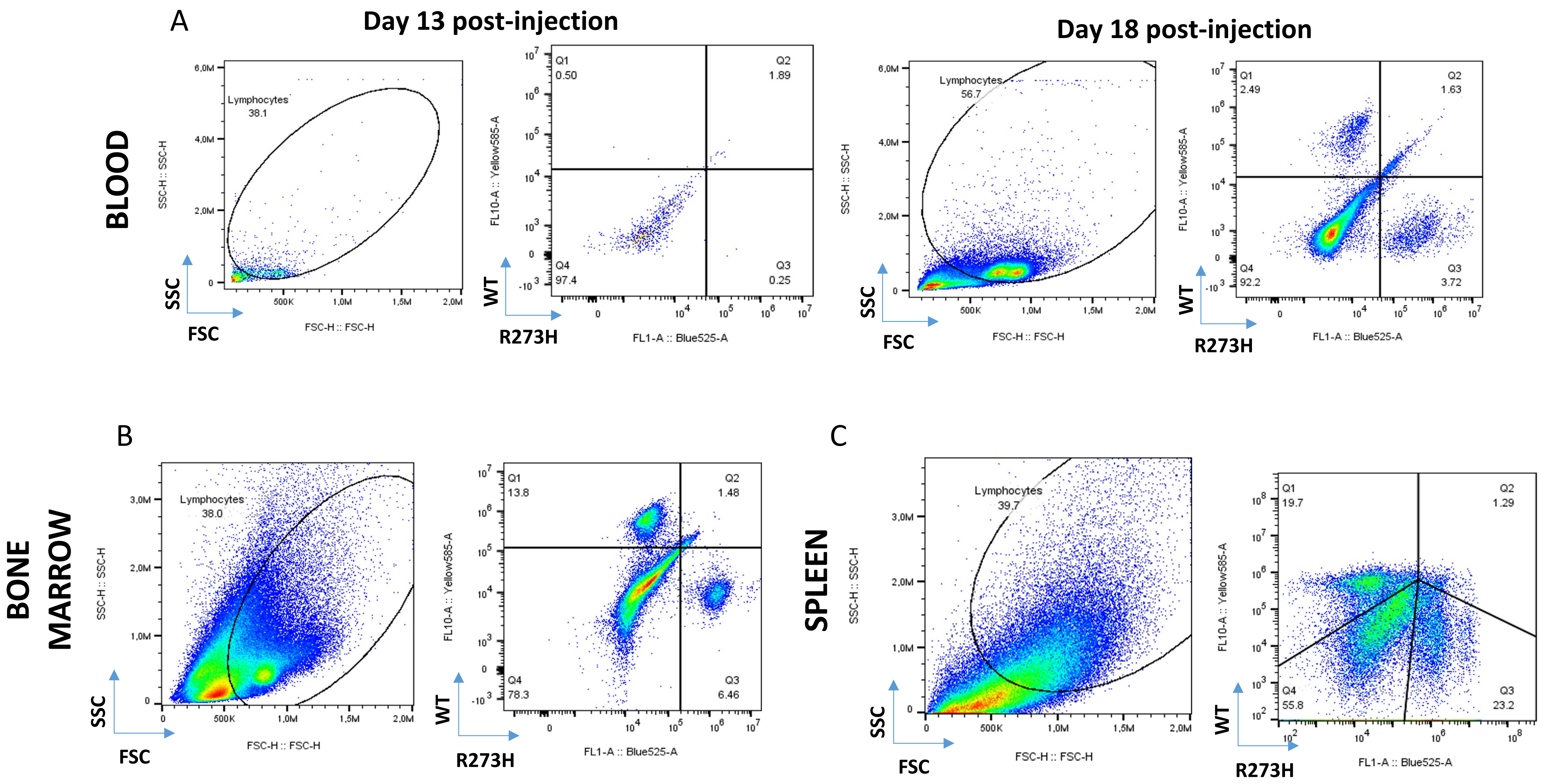

Supplementary Figure legends

Figure 1: **Inhibitory concentration of isogenic MOLM-13 and K562 cell lines TP53 WT or mutated to Azacitidine (AZA) and Niclosamide. A**, **B** Inhibitory concentrations to Niclosamide and AZA in (**A)** MOLM-13 and (**B)** K562 TP53 wild-type (WT), KO or harboring the R248Q, R273H and R175H. **C**, **D** Dose effect curves to AZA (**C**) alone and (**D**) after addition of the IC20 of Niclosamide to each cell line. *: $p<0.05$, **: $p<0.01$, ***: $p<0.001$, ****: $p<0.0001$.

Figure 2: **Ex-vivo visualization of MOLM-13 cells by FACS analysis. A, B, C** Gating strategy for tracking MOLM-13 cells TP53 WT-mCherry or R273H-GFP by FACS analysis in (**A**) Blood at Day 13 post-injection and Day 18 post-injection as mentioned (**B**) Bone Marrow at sacrifice at day 19 and (**C**) Spleen at sacrifice at day 19.
